# Clinical Significance of Tumor Necrosis Factor-α Inhibitors in the Treatment of Sciatica: A Systematic Review and Meta-Analysis

**DOI:** 10.1371/journal.pone.0103147

**Published:** 2014-07-22

**Authors:** Yun Fu Wang, Ping You Chen, Wei Chang, Fi Qi Zhu, Li Li Xu, Song Lin Wang, Li Ying Chang, Jie Luo, Guang Jian Liu

**Affiliations:** 1 Department of Neurology, Taihe Hospital Affiliated to Hubei University of Medicine, Shiyan City, Hubei Province, China; 2 Medical Imaging Center, Taihe Hospital Affiliated to Hubei University of Medicine, Shiyan City, Hubei Province, China; 3 Department of Spine Surgery, Taihe Hospital Affiliated to Hubei University of Medicine, Shiyan City, Hubei Province, China; 4 Department of Neurology, Yuebei People’s Hospital Affiliated to Shantou University Medical College, Shaoguan City, Guangdong Province, China; 5 Department of Neurology, Xiangyang Center Hospital Affiliated to Hubei University of Arts and Science, Xiangyang City, Hubei Province, China; Emory University, United States of America

## Abstract

**Background and Objective:**

Currently, no satisfactory treatment is available for sciatica caused by herniated discs and/or spinal stenosis. The objective of this study is to assess the value of tumor necrosis factor (TNF)-α inhibitors in the treatment of sciatica.

**Methods:**

Without language restrictions, we searched PubMed, OVID, EMBASE, the Web of Science, the Clinical Trials Registers, the Cochrane Central Register of Controlled Trials and the China Academic Library and Information System. We then performed a systematic review and meta-analysis on the enrolled trials that met the inclusion criteria.

**Results:**

Nine prospective randomized controlled trials (RCTs) and two before-after controlled trials involving 531 patients met our inclusion criteria and were included in this study. Our systematic assessment and meta-analysis demonstrated that in terms of the natural course of the disease, compared with the control condition, TNF-α inhibitors neither significantly relieved lower back and leg pain (both p>0.05) nor enhanced the proportion of patients who felt overall satisfaction (global perceived effect (satisfaction)) or were able to return to work (return to work) (combined endpoint; p>0.05) at the short-term, medium-term and long-term follow-ups. In addition, compared with the control condition, TNF-α inhibitors could reduce the risk ratio (RR) of discectomy or radicular block (combined endpoint; RR = 0.51, 95% CI 0.26 to 1.00, p = 0.049) at medium-term follow-up, but did not decrease RR at the short-term (RR = 0.64, 95% CI 0.17 to 2.40, p = 0.508) and long-term follow-ups (RR = 0.64, 95% CI 0.40 to 1.03, p = 0.065).

**Conclusion:**

The currently available evidence demonstrated that other than reducing the RR of discectomy or radicular block (combined endpoint) at medium-term follow-up, TNF-α inhibitors showed limited clinical value in the treatment of sciatica caused by herniated discs and/or spinal stenosis.

## Introduction

Disk herniation-induced sciatica is one of the most common causes of lower back and leg pain among young adults. Previous studies have demonstrated that the outcomes of conservative treatment, such as medication and physical therapy, are similar to the natural course of this disease [1]. Although epidural steroid injections can relieve a portion of patients’ pain, they cannot restore the patients’ physical function [2]. Recently, some scholars have stated that non-opioid analgesic agents, discectomy and epidural steroid injection are effective [3]; however, the opposing opinion indicates that discectomy is only effective for acute neurodynia, and its long-term outcome is not superior to that of conservative treatment [4]. In addition, because of nerve root adhesions or epidural adhesions, epidural steroid injection cannot relieve pain in a considerable number of patients [5].

Tumor necrosis factor-alpha (TNF-α) is an inflammatory factor involved in the pathophysiological mechanism underlying disk herniation-induced sciatica [6], [7]. In the past decade, some scholars have attempted to use TNF-α inhibitors to treat sciatica. Previous non-randomized controlled trials have shown that this type of agent has potential efficacy and a relatively high patient tolerance [8], [9]. However, afterwards, various randomized controlled trials (RCTs) demonstrated that these agents yielded inconsistent outcomes. A newly published systematic review and meta-analysis revealed that the evidence supporting the use of TNF-α inhibitors to treat sciatica is inadequate [10]. Nevertheless, this study has some limitations: (1) four high-quality RCTs [11]–[14] were missed; (2) among all of the enrolled trials, a visual analogue scale (VAS) score range of 0 to 100 was adopted in a portion of trials [15]–[19], while a score range of 0 to 10 was applied in others [20]–[22]. The authors used a weighted mean difference (WMD) technique to pool all of the data together; however, this is not a standard and conventional method commonly used in meta-analysis [23]; and (3) in addition, we disagree that the authors’ method of pooling together all of the data regarding the outcomes of discectomy, including the data obtained during short-term, medium-term and long-term follow-ups.

The primary purpose of this study was to evaluate the treatment value of TNF-α inhibitors compared with placebos and steroids in terms of five endpoints at short-term follow-up (≤3 months), medium-term follow-up (3 to 12 months) and long-term follow-up (≥12 months). The five endpoints that were adopted were the Oswestry Disability Index, VAS pain intensity in the leg, VAS pain intensity in the lower back, global perceived effect (satisfaction) or return to work (combined endpoint), and discectomy or radicular block (combined endpoint). The secondary purpose was to evaluate the patient tolerance of the adverse reaction of TNF-α inhibitors.

## Methods

Using the “Preferred Reporting Items for Systematic reviews and Meta-Analyses (PRISMA)” [24] as a guideline, we conducted this systematic review and meta-analysis. The present study is a complement to and update of the study performed by Williams *et al.* [10].

### Search Strategies

The searched database included the following: PubMed, OVID, EMBASE, the Web of Science, the Clinical Trials Registers, the Cochrane Central Register of Controlled Trials and the China Academic Library and Information System. The search terms included following: “anti-tumor necrosis factor agents OR tumor necrosis factor alpha inhibitor OR infliximab OR adalimumab OR etanercept OR rituximab OR golimumab OR certolizumab OR efalizumab OR ustekinumab OR alefacept” AND “sciatica OR lumbosacral radiculopathy” AND “controlled trial” appearing in “title/abstract”. Each database was searched from January 1, 2000 to July 1, 2013. No language restrictions were applied.

### Trial Selection

The inclusion criteria were as follows: (1) Participants: all patients included were older than 18 years and were diagnosed with sciatica caused by lumbar disc herniation and/or lumbar spinal stenosis confirmed with CT/MRI, regardless of the duration of symptoms. Patients who planned to undergo discectomy soon or had comorbid liver disease, tuberculosis, spinal cord tumor, infection or trauma were excluded; (2) Intervention: any trial that used TNF-α inhibitors in the TNF-α inhibitor group and placebos or steroids in the control group and in which all drugs were locally injected or systematically administered; (3) Endpoints: any trial that used subjective parameters, such as the Oswestry Disability Index and VAS scores, to evaluate lower back and leg pain and used global perceived effect (satisfaction) or return to work (combined endpoint) to represent the proportion of patients who felt overall satisfaction or were able to return to work, and adopted an objective parameter, discectomy or radicular block (combined endpoint), to evaluate the risk ratio (RR) of discectomy or radicular block; (4) Study type: the controlled trials including randomized controlled trials (RCTs), cross-over controlled trial, non-randomized concurrent trials, before-after controlled trial, and case-control study, were included regardless of their sample size and trial results.

### Data Extraction

Using a unified form, two investigators extracted the data and established the data spreadsheet independently. Finally, they confirmed the accuracy of the data together, and discrepancies were resolved via discussion until a consensus was reached. A portion of the endpoint data expressed only as a line graph or histogram was obtained from the forest plots of the study conducted by Williams *et al.* [10]. The extracted data mainly included the sample size, intervention measures, the Oswestry Disability Index, VAS-leg pain, VAS-lower back pain, global perceived effect (satisfaction) or return to work (combined endpoint), and discectomy or radicular block (combined endpoint) of the experimental and control groups at various follow-up points.

### Quality Evaluation

One investigator performed a methodology quality assessment of all included studies based on a 17-item quality evaluating system [25].

### Statistical Analysis

Using WMD, standardized mean difference (SMD) and RR, we performed a systematic review and meta-analysis of the aforementioned five endpoints according to the follow-up time and the type of control drugs used. For global perceived effect (satisfaction) or return to work (combined endpoint), an RR>1 indicated that the outcomes of the TNF-α inhibitor group were superior to those of the control group; for discectomy or radicular block (combined endpoint), an RR<1 indicated that the outcomes of the TNF-α inhibitor group were superior to those of the control group; for Oswestry Disability Index, VAS-leg, and VAS-lower back, a negative WMD or SMD indicated that the outcomes of the TNF-α inhibitor group were superior to those of the control group. The data from reports concerning same trial were used for the analysis of the corresponding follow-up. Prior to the meta-analysis, for each endpoint, Cochran’s Q statistic test was applied to assess the heterogeneity among the included studies. If a p-value of Cochran’s Q statistic (Qp) ≥0.10, which indicated the absence of heterogeneity, a fixed-effects model was applied; otherwise, a random effects model was applied for analysis. Stata statistical software version SE 12.0 (Stata Corp LP, College Station, TX, USA) was utilized for all statistical analysis.

## Results

### Search Results

**Figure 1:**
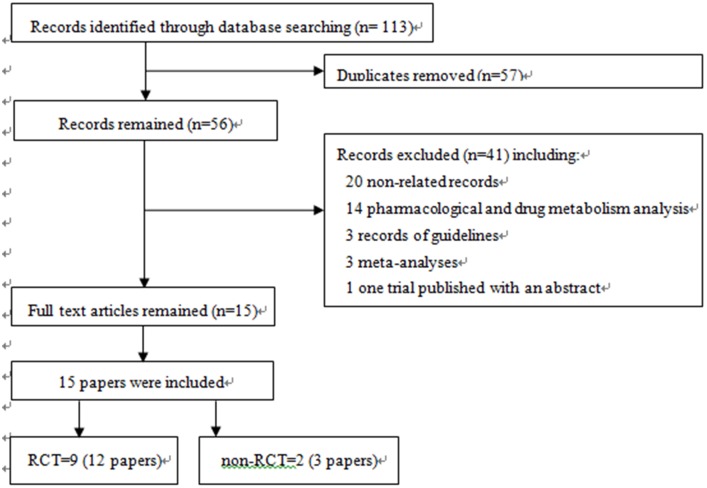
A flow diagram of the screening process.

A total of 113 records were identified through database searches, and 16 remained [8], [9], [11]–[22], [26], [27] after the exclusion of unrelated and repeated studies through a careful review of the titles, abstracts and partial main text. After the exclusion of one trial that was published as an abstract without full text available [26], 15 papers were ultimately enrolled in our study [8], [9], [11]–[22], [27]. These 15 papers included nine RCTs [11]–[22], [27], two non-RCT (before-after controlled trials) [8], [9], [27], involving 531 patients; two of the trials were reports of the data from the 6-month and 36-month follow-up of the NCT00470509 trial [13], [15], three of the trials were the reports of the data from the 3-month, 6-month and 12-month follow-ups of the FIRST II trial [11], [16], [19], and two records were the reports of the data from the 3-month, 6-month and 12-month follow-ups of same trial [9], [27]. Among the included trials, seven used placebos as a control [9], [14]–[17], [20], [22], three used steroids as a control [8], [12], [18], and one used placebos and steroids as a dual control [21]; eight trials involved local injection [8], [12]–[14], [18], [20]–[22], and three involved a systematic medication [9], [16], [17]; the drugs were administered once in seven trials [9], [12], [14], [16], [17], [22], twice in three trials [13], [20], [21], and three times in two trial [8], [18]; six trials adopted VAS scoring in a range of 0 to100 [8], [9], [15]–[18], and five adopted VAS scoring in a range of 0 to 10 [12], [14], [20]–[22]. Figure 1 shows the screening process. The major features of the 11 trials are listed in Table 1.

**Table 1:** Trial characteristics.

Trial and studytype		Participants	Intervention		Outcome (follow-up duration andoutcome measures)
			TNF-α inhibitorgroup	Control group	
1	Genevay *et al.* [13], [15], RCT	n = 61, and theaverage age was49 years; malesaccounted for 57%of the participants;the mean durationof the symptomswas 3.6 weeks	Adalimumab40 mg;subcutaneousinjection;once a week;administeredtwice	Normal saline;subcutaneous injection;once a week;administered twice	Genevay *et al.* [15]: 6-week and 6-monthfollow-ups; VAS-leg pain, VAS-lowerback pain, Oswestry Disability Index [28],the number of resected discs, thegeneral health survey (12-item Short Form health survey [29]), the number of patients who returned to work, adverse reaction
					Genevay *et al.* [13]: 36-month follow-up;The number of resected discs, VAS-leg pain,VAS-lower back pain, Oswestry DisabilityIndex [28], the general health survey(12-item Short Form health survey [30])
2	Cohen *et al.* [21], RCT	n = 84, and theaverage age was 42 years;males accounted for 70%of the population;the mean duration ofthe symptoms was 2.7 months	Etanercept 4 mg+bupivacaine0.5 ml;foraminalinjectionadministeredtwice	Normal saline + bupivacaine0.5 ml (Group 1),or steroid methylprednisolone 60 mg +bupivacaine 0.5 ml(Group 2); foraminalinjection administered twice	1-, 3- and 6-month follow-ups; Thenumber of patients with a positiveoutcome (a 50% reduction in leg pain + anoverall positive feeling without any furthertreatment), VAS-leg pain, VAS-lower backpain, Oswestry Disability Index [28],complications
3	Ohtori *et al.* [12], RCT	n = 80, and the averageage was65±5.5/67±5.0^1^years; the meanduration of thesymptoms was 2.5(1 to 12)/2.3 (1 to 12)^2^months	Etanercept 10 mg;foraminal injection;administered once	Dexamethasone 3.3 mg;foraminal injection; administered once	1-month follow-up; VAS-leg pain,VAS-lower back pain, Oswestry DisabilityIndex [28], complications
4	Okoro *et al.* [22], RCT	n = 15 with noindication ofthe average age;males accounted for40% of thepopulation; theduration of thesymptoms was atleast 24 weeks	Etanercept 25 mg;subcutaneousinjection; administeredonce	Normal saline;subcutaneousinjection; once	3-month follow-up; VAS-leg pain,VAS-lower back pain, OswestryDisability Index [28], modified Zung Depression index, independent walking distance, the number of patients receiving discectomy or radicular block, adverse reaction
5	Cohen *et al.* [20], RCT	n = 24, and the medianage was 41 to 46 years;males accounted for 71% of theparticipants; the meanduration of symptomswas 3 to 7 months	Etanercept 2 mg(Group 1), 4 mg(Group 2), 6 mg(Group 3); foraminalinjection; administeredonce	Normal saline; foraminalinjection; administeredonce	1-, 3- and 6-month follow-ups; VAS-leg pain,VAS-lower back pain, Oswestry DisabilityIndex [28], the number of patients witha positive outcome (a reduction of 50%in the leg pain + an overall satisfaction),the number of resected discs
6	Karppinen *et al.* [17], RCT	n = 15, and the average agewas 53 years; malesaccounted for 67%of the participants;the mean durationof symptoms was 58days	Infliximab 5 mg/kg;intravenous injection;administered once	Normal saline;intravenous injection; administeredonce	3- and 6-month follow-ups; VAS-leg pain,VAS-lower back pain, Oswestry DisabilityIndex [28], the number of patients whounderwent discectomy or caudal epiduralblock, RAND-36-item health questionnaire,days of sick leave, adverse reaction
7	Cohen *et al.* [14], RCT	n = 36, and the averageage was 39.3±1.9^1^years; malesaccounted for 78%of the participants;the mean durationof the symptomswas 5.3±0.7^1^ years	Etanercept 0.1 mg(Group 1), 0.5 mg(Group 2), 0.75 mg(Group 3), 1.0 mg(Group 4), 1.5 mg(Group 5); subcutaneousinjection; administeredonce	Normal saline;subcutaneousinjection; administeredonce	1-, 3-and 6-month follow-ups; VAS-legpain, VAS-lower back pain, Oswestry DisabilityIndex [28], overall satisfaction score
8	Becker *et al.* [18], RCT	n = 84, and the averageage was 54 years;males accountedfor 62% of theparticipants; theduration of thesymptoms was atleast 6 weeks	Autologous conditionedserum (Group 1);epiduralinjections;administeredthree times	Triamcinolone 5 mgor 10 mg + localanesthetic 1 ml(Group 2 or Group 3);epidural injection;administered three times	6-, 10- and 22-week follow-ups; VAS-lowerback pain, Oswestry Disability Index [28],adverse reaction
9	Korhonen *et al.* [11], [16], [19], RCT	n = 40, and the averageage was 40 years;the males accountedfor 60%; the meanduration of the symptomswas 61 days	Infliximab 5 mg/kg;intravenous injection;administered once	Normal saline;intravenousinjection;administeredonce	Korhonen *et al.* [19]: 3-month follow-up;The straight leg-raising test, VAS-leg pain,VAS-lower back pain, Oswestry DisabilityIndex [28]
					Autio *et al.* [11]: 6-month follow-up;The volume (mm^3^), thickness (mm) and therim enhancement (%) of herniated nucleuspulposus, the number of resected discs, swelling of the nerve root
					Korhonen *et al.* [16]: 12-month follow-up; The straight leg-raising test, VAS-leg pain, VAS-lower back pain, Oswestry Disability Index [28], RAND-36-item health questionnaire [31], the number of resected discs, adverse reaction
10	Karppinen *et al.* [9]and Korhonen *et al.* [27], non-RCT	n = 72, and the averageage was 39 years; themales accounted for80%; the meanduration of thesymptoms was 7.2 weeks	Infliximab 3 mg/kg;intravenous injection;administered once	Normal saline;Periradicularinjection;administeredonce	Karppinen *et al* [9]: 3-month follow-up;The number of painless patients (75% decrease from baseline leg pain score), VAS-leg pain
					Korhonen *et al* [27]: 6- and 12-monthfollow-ups; VAS-leg pain, VAS-lower backpain, Oswestry Disability Index [28],the number of sick leave days; clinical status,adverse effects
11	Genevay *et al.* [8],non-RCT	n = 20, and the averageage was 47 years;the males accountedfor 50%; the meanduration of thesymptoms was 3.2 weeks	Etanercept 25 mg;subcutaneousinjection; every 3 days;administered three	Methylprednisolone;250 mg, intravenousinjection;administered three	6-weeks follow-up; The numbers witha good clinical result (leg pain VAS<30 or Oswestry Disability Index<20); VAS-leg pain, VAS-lower back pain, Oswestry Disability Index [28]; Roland Morris Disability Questionnaire (RMDQ) [32], the number of discectomies

RCT randomized controlled trial, non-RCT non-randomized control trial, VAS visual analogue scale.

1 mean ± standard deviation.

2 median (range).

### Quality of all included studies

Table 2 lists the quality scores of the 11 trials, including one with high quality [21], nine with middle quality (12 records) [8], [11]–[20], [22], and one with poor quality (two records) [9], [27].

**Table 2:** Methodological quality scoring [25] for all trials.

Check list item	Trials										
	1 [13], [15]	2 [21]	3 [12]	4 [22]	5 [20]	6 [17]	7 [14]	8 [18]	9 [11], [16], [19]	10 [9], [27]	11 [8]
1. Is there a rationale for the study?	yes	yes	yes	yes	yes	yes	yes	yes	yes	yes	yes
2. Is a clear study objective/goal defined?	yes	yes	yes	yes	yes	yes	yes	yes	yes	no	yes
3. Are key elements of study design described (e.g. howwere participants identified/recruited)?	yes	yes	yes	yes	yes	yes	yes	yes	yes	no	yes
4. Are the setting and selection criteria for the studypopulation described?	yes	yes	yes	yes	yes	yes	yes	yes	yes	yes	yes
5. Is the follow-up period appropriate?	yes	yes	no	yes	yes	yes	yes	yes	yes	yes	no
6. Are there any strategies to avoid loss to follow-upor address missing data?	no	no	no	no	no	no	no	no	no	no	no
7. Is the sample size justified?	no	no	no	no	no	no	no	no	no	no	no
8. Is information presented about the instruments usedto measure the prognostic variable(s), and does thisenable replication (through the use ofstandardized or valid measures)?	yes	yes	yes	yes	yes	yes	yes	yes	yes	yes	yes
9. Is the outcome selected and assessed appropriately?	yes	yes	yes	yes	yes	yes	yes	yes	yes	yes	yes
10. Is the study sample described (demographic/clinicalcharacteristics)?	yes	yes	yes	no	yes	yes	yes	no	yes	yes	yes
11. Is the final sample representative of the study’starget population?	yes	yes	yes	yes	no	yes	yes	yes	yes	no	no
12. Is loss to follow-up ≤20%? (If not, are there any significantdifferences in baseline variables between responders andnon-responders to follow-up? If yes, have the implications been considered?)	yes	yes	yes	yes	no	yes	yes	yes	yes	yes	yes
13. Are the main results reported (including the prevalence ofprognostic indicator(s) and outcome, strength of association,and statistical significance)?	yes	yes	yes	yes	yes	yes	yes	yes	yes	yes	yes
14. Is the statistical analysis appropriate and described?	yes	yes	yes	no	yes	yes	yes	yes	yes	yes	yes
15. Were potential confounders and effect modifiersidentified and accounted for (e.g. multivariate analysis)?	yes	yes	yes	yes	yes	yes	yes	yes	yes	no	no
16. Do the findings support the authors’ interpretations?	yes	yes	yes	yes	yes	yes	yes	yes	yes	yes	yes
17. Do the authors discuss studylimitations (e.g. biases/generalizability)?	no	yes	yes	no	yes	no	no	no	no	no	no
Total	14	15	14	12	13	14	14	13	14	10	11

Scoring: Total number of “yes” answers provides the overall score. 0 to 10 = poor quality, 11 to 14 = adequate quality, 15 to 17 = high quality [25].

### Oswestry Disability Index

The trial by Cohen *et al.* [20] demonstrated there was no statistical difference in the Oswestry Disability Index between the two groups at post-injection Month 1 and Month 6 (p = 0.11, p = 0.78). Another trial by Cohen *et al.* [14] showed that there was no intragroup or intergroup difference in the Oswestry Disability Index at post-injection Month 1, while the Oswestry Disability Index was restored to the baseline level at post-injection Month 6. The trial conducted by Karppinen *et al.* [17] also indicated that there was no statistical difference in the Oswestry Disability Index between the two groups at post-injection Month 6 (p = 0.52). In addition, in the FIRST II trial, there was no statistical difference in the Oswestry Disability Index between the two groups at post-injection Month 3 and Month 12 (p = 0.37, p = 0.79) [16], [19].

**Figure 2:**
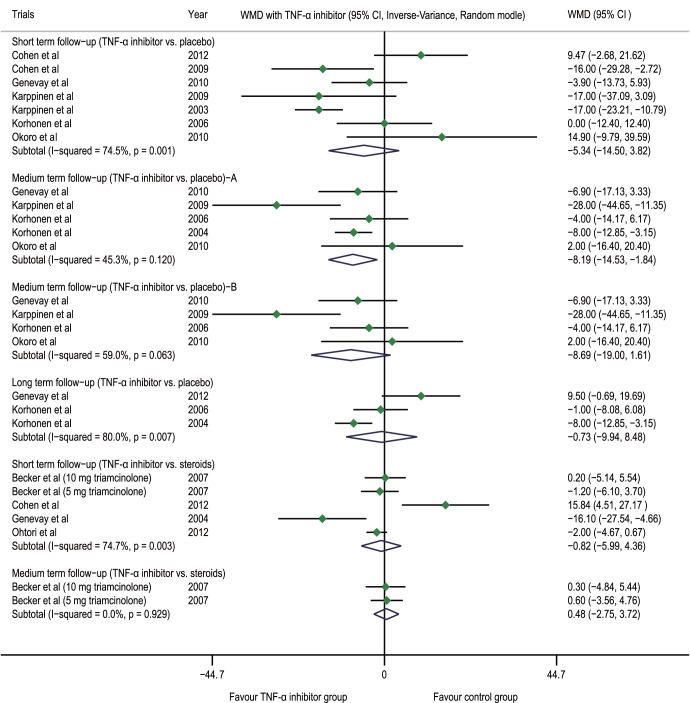
Forest plot of Oswestry Disability Index. The difference in the Oswestry Disability Index (WMD) at the post-injection short-term, medium-term and long-term follow-ups between the TNF-α inhibitor group and placebo group was not statistically significant (p>0.05); there was no statistically significant difference at the post-injection short-term and medium-term follow-ups between the steroid group and the TNF-α inhibitor group (p>0.05). A. Results when the trials with poor quality were included; B. Results when the trials with poor quality were excluded. TNF-α tumor necrosis factor-alpha, CI confidence interval, WMD weighted mean difference.

The results derived from the meta-analysis showed that compared with the placebo group, the TNF-α inhibitor group had a WMD of −5.34 (−14.50 to 3.82, p = 0.254, n = 7) at the post-injection short-term follow-up, −8.19 (−14.53 to −1.84, p = 0.011, n = 5) at the medium-term follow-up (The sensitivity analysis demonstrated that after the exclusion of a low-quality trial [27], WMD = 8.69, 95% CI 19.00 to 1.61, p = 0.098, n = 4), and −0.73 (−9.94 to 8.48, p = 0.877, n = 3) at the long-term follow-up; compared with the steroid group, the TNF-α inhibitor group had a WMD of −0.82 (−5.99 to 4.36, p = 0.757, n = 5) at the post-injection short-term follow-up and 0.48 (−2.75 to 3.72, p = 0.771, n = 2) at the medium-term follow-up (Figure 2).

### VAS-leg pain

The trial by Cohen *et al.* [20] showed no statistical difference in the reduction of VAS between the two groups at post-injection Month 1 (p = 0.15). Another trial by Cohen *et al.* [14] indicated there was no difference in the VAS between the two groups at post-injection Month 1 [14]. The trial by Karppinen *et al.* [17] showed no statistical difference in the reduction of leg pain between the two groups at post-injection Month 6 (73% vs. 65%, p = 0.52). In addition, in the FIRST II trial, there was no statistical difference observed in the VAS-leg pain between the two groups at post-injection Month 3 and Month 12 (p = 0.82, p = 0.54) [16], [19]; moreover, there was no statistical difference in the percentage of patients who achieved a VAS reduction of more than 75% between the two groups at post-injection Month 12 (p = 0.72) [16].

**Figure 3:**
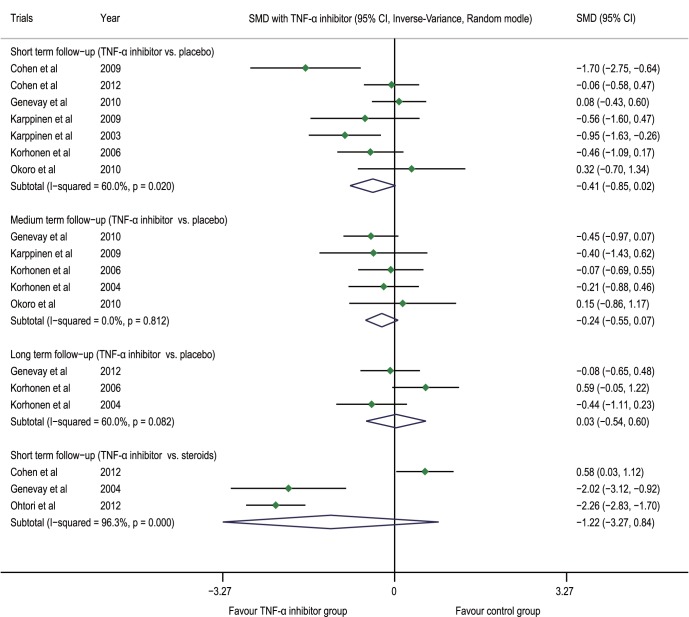
Forest plot of VAS-leg pain. The difference in the VAS-leg (SMD) at the post-injection short-term, medium-term and long-term follow-ups between the TNF-α inhibitor group and placebo group was not statistically significant (p>0.05); there was no statistically significant difference at the post-injection short-term follow-up between the steroid group and the TNF-α inhibitor group (p>0.05). VAS visual analogue scale, TNF-α tumor necrosis factor-alpha, CI confidence interval, SMD standardized mean difference.

The meta-analysis showed that, compared with the placebo group, the TNF-α inhibitor group had an SMD of −0.41 (−0.85 to 0.02, p = 0.061, n = 7) at the post-injection short-term follow-up, −0.24 (−0.55 to 0.07, p = 0.122, n = 5) at the medium-term follow-up, and 0.03 (−0.54 to 0.60, p = 0.928, n = 3) at the long-term follow-up; compared with the steroid group, the TNF-α inhibitor group had an SMD of −1.22 (−3.27 to 0.84, p = 0.246, n = 3) at the post-injection short-term follow-up (Figure 3).

### VAS-lower back pain

**Figure 4:**
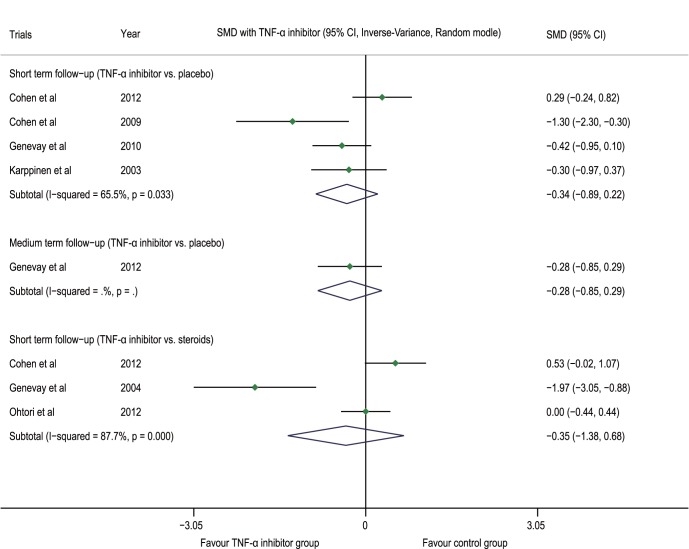
Forest plot of VAS-lower back pain. The difference in the VAS-lower back (SMD) at the post-injection short-term and medium-term follow-ups between the TNF-α inhibitor group and placebo group was not statistically significant (p>0.05); there was no statistically significant difference at the post-injection short-term follow-up between the steroid group and the TNF-α inhibitor group (p>0.05). VAS visual analogue scale, TNF-α tumor necrosis factor-alpha, CI confidence interval, SMD standardized mean difference.

A trial by Cohen *et al.* [20] showed that the VAS-lower back pain of the TNF-α inhibitor group was significantly lower than that of the placebo group (p = 0.01) one month after drug injection. The trial by Karppinen *et al.* [17] showed no statistical difference in the VAS between the two groups at post-injection Months 3 and 6 (p = 0.13, p = 0.25). In addition, in the FIRST II trial, there was no statistical difference observed in the VAS-lower back pain between the two groups at post-injection Month 3 and Month 12 (p = 0.98, p = 0.68) [16], [19]. The meta-analysis demonstrated that compared with the placebo group, the TNF-α inhibitor group had an SMD of −0.34 (−0.89 to 0.22, p = 0.233, n = 4) at the post-injection short-term follow-up and −0.28 (−0.85 to 0.29, p = 0.332, n = 1) at the medium-term follow-up; compared with the steroid group, the TNF-α inhibitor group had an SMD of −0.35 (−1.38 to 0.68, p = 0.503, n = 3) at the post-injection short-term follow-up (Figure 4).

### Global perceived effect (satisfaction) or return to work (combined endpoint)

**Figure 5:**
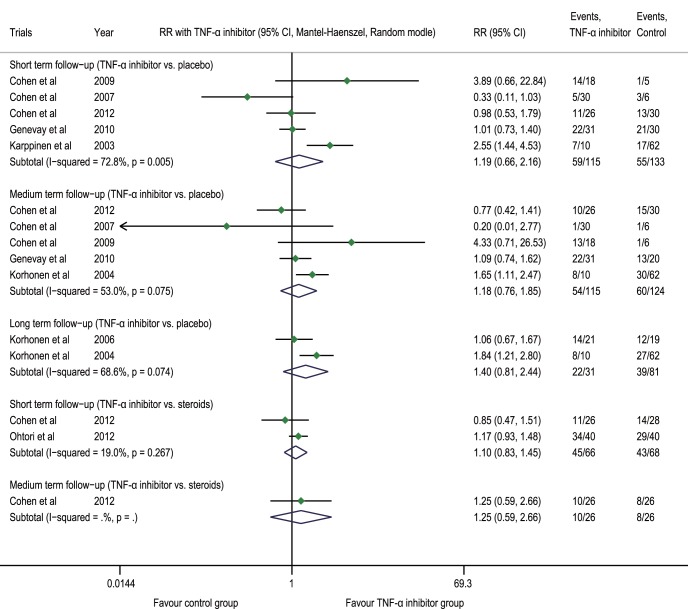
Forest plot of global perceived effect (satisfaction) or return to work (combined endpoint). The difference in the RR of global perceived effect (satisfaction) or return to work (combined endpoint) at the post-injection short-term, medium-term, and long-term follow-ups between the TNF-α inhibitor group and placebo group was not statistically significant (p>0.05); there was no statistically significant difference at the post-injection short-term and medium-term follow-ups between the steroid group and the TNF-α inhibitor group (p>0.05). TNF-α tumor necrosis factor-alpha, CI confidence interval, RR risk ratio.

The trial by Genevay *et al.* [13] demonstrated no difference in the work capability or physical condition between the two groups, and the trial by Cohen *et al.* [14] also indicated no intragroup and intergroup difference in the patients’ global perceived effect. In the FIRST II trial, no statistical difference was observed between the two groups in the patients’ days of sick leave from work because of sciatica (p = 0.60) [16]. The meta-analysis indicated that compared with the placebo group, the TNF-α inhibitor group had an RR of 1.19 (0.66 to 2.16, p = 0.554, n = 5) for global perceived effect (satisfaction) or return to work (combined endpoint) at the post-injection short-term follow-up, 1.18 (0.76 to 1.85, p = 0.465, n = 5) at the medium-term follow-up and 1.40 (0.81 to 2.44, p = 0.231, n = 2) at the long-term follow-up; compared with the steroid group, the TNF-α inhibitor group had an RR of 1.10 (0.83 to 1.45, p = 0.520, n = 2) at the short-term follow-up and 1.25 (0.59 to 2.66, p = 0.562, n = 1) at the medium-term follow-up (Figure 5).

### Discectomy or radicular block (combined endpoint)

**Figure 6:**
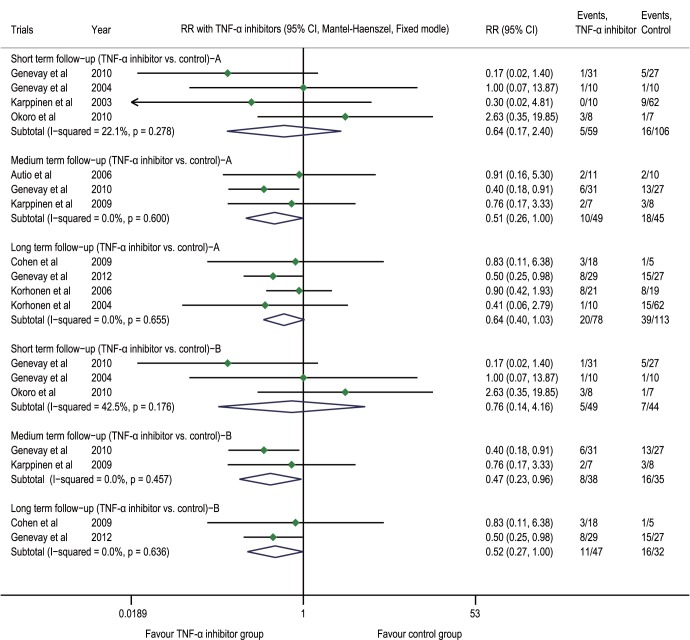
Forest plot of discectomy of the radicular block (combined endpoint). The difference in the RR of discectomy or the radicular block (combined endpoint) at the post-injection short-term and long-term follow-ups between the TNF-α inhibitor group and placebo group was not statistically significant (p>0.05). At the medium-term follow-up, the RR of the TNF-α inhibitor group was 66% of that of the placebo group; after the exclusion of the three trials involving a systemic medication, the RR of the TNF-α inhibitor group was 47% of that of the placebo group at the medium-term follow-up, and was 52% of that of the placebo group at the long-term follow-up. A. Results when the trials involving a systemic medication were included; B. Results when the trials involving a systemic medication were excluded. TNF-α tumor necrosis factor-alpha, CI confidence interval, RR risk ratio.

The results derived from the meta-analysis indicated that compared with the control group, the TNF-α inhibitor group had an RR of 0.64 (0.17 to 2.40, p = 0.508, n = 4) for the discectomy or radicular block (combined endpoint) at the post-injection short-term follow-up, 0.51 (0.26 to 1.00, p = 0.049, n = 3) at the medium-term follow-up and 0.64 (0.40 to 1.03, p = 0.065, n = 4) at the long-term follow-up (Figure 6A). The sensitivity analysis showed that after the exclusion of the trials with a systematic drug administration [11], [16], [27], the RR was 0.47 (0.23 to 0.96, p = 0.037, n = 2) at the medium-term follow-up and 0.52 (0.27 to 1.00, p = 0.049, n = 2) at the long-term follow-up (Figure 6B).

### Adverse reaction

Four reports indicated the absence of adverse reaction [11], [12], [19], [22], six reports did not mention adverse reaction [8],[9],[13],[14],[20],[27], and four reported the occurrence of rhinitis, diarrhea, otitis media, maxillary sinusitis or a skin rash, but with only “mild symptoms” [15]–[17], [21]. One patient had metastasis of cancer cells at the neck [17], and one patient had severe digestive tract bleeding after being treated with non-steroidal anti-inflammatory agents [15]. One trial reported the occurrence of puncture-associated severe headache in both groups [18]. However, there was no evidence proving the relationship between these adverse events and the use of TNF-α inhibitors.

## Discussion

The major findings of this study were that TNF-α inhibitors could reduce the RR of discectomy or radicular block (combined endpoint) at medium-term follow-up, but not at short-term and long-term follow-ups; during the natural disease course, TNF-α inhibitors neither provided additional pain relief nor improved the percentage of global perceived effect (satisfaction) or return to work (combined endpoint); the observed adverse reaction were mild and could not be proven to have any association with the use of TNF-α inhibitors.

Previous studies have found that in patients with intervertebral disc herniation, the TNF-α level on the articular surface increased [33]; the TNF-α mRNA level on the annulus fibrosus, nucleus pulposus and the yellow ligaments increased [34]; and the content of nucleus pulposus leaked to the epidural cavity, causing local acute inflammation, fiber deposition and adhesion [5]. The inflammatory reactions mediated by various biochemical and immunological factors can disturb intraradicular blood flow and disrupt the nerve-blood barrier, thus leading to swelling and demyelination of the nerve root [35]. TNF-α is a pleiotropic cytokine, which can induce inflammatory responses of synapses and myelin sheath [35], promote cellular apoptosis due to its cytotoxic effect [6], and induce nerve swelling and neuropathic pain [35]. Thus, this cytokine plays a pivotal role in the pathophysiology of sciatica [36]. Animal experiments and clinical studies have revealed that TNF-α inhibitors can prevent the decline of nucleus pulposus-induced neural transmission speed and neural damage and thus have a protective effect on neurodegeneration [7], [37], [38]. The results from clinical trials in terms of the efficacy of TNF-α inhibitors on decreasing the RR of discectomy or radicular block (combined endpoint) are inconsistent; a portion of the trials showed positive results [13], [15], whereas others reported that TNF-α inhibitors had efficacy similar to that of placebos [8], [9], [11], [12], [14], [16]–[22], [27]. In this study, we found that TNF-α inhibitors could decrease the RR of discectomy or radicular block (combined endpoint) at the medium-term follow-up. We conservatively speculate that this effect might be related to their anti-inflammatory and neuroprotective functions.

Our explanations regarding the finding that TNF-α inhibitors could reduce the risk of discectomy or radicular block (combined endpoint) but could not improve the pain were as follows: (1) TNF-α inhibitors reduce the risk of discectomy or radicular block (combined endpoint) because of their neuroprotective function. Genevay *et al.* [13], [15] believe that TNF-α inhibitors might have a “delayed mode of action on sciatica” or protective function on the nerve root, and thus the physical condition could recover better with TNF-α inhibitors; this result should be considered during the selection of discectomy. The trial by Korhonen *et al.* [27] showed that the recovery rate of nerve abnormalities was much higher in the TNF-α inhibitor group than in the control group (p = 0.001), indirectly indicating that TNF-α inhibitors had a protective effect on sciatic nerves. Because of this neuroprotective effect, TNF-α inhibitors can promote the recovery of physical function and reduce the risk of discectomy or radicular block (combined endpoint). (2) The anti-inflammatory effect of TNF-α inhibitors is not parallel to their pain-controlling effect. The mechanisms underlying leg pain and lower back pain remain unclear [39]. Scholars generally believe the pain might result from the mechanical, chemical and inflammatory irritation of sinuvertebral nerves [40], [41], while the pain might be associated with unstable lumbar spine or spinal stenosis in addition to inflammatory responses in a portion of patients. The trial by Andrade *et al.* [42] found that in the patients with disc herniation, the expression of TNF-α, IL-1β and IL-6 increased significantly, but the expression levels of these cytokines were unrelated to the lower back pain; therefore, the authors concluded that “these cytokines may not play a leading role in maintaining a pain generating network”, indicating that TNF is not the sole inflammatory factor and TNF-mediated inflammatory response is not the leading cause responsible for the pain of disc herniation patients. Moreover, some studies demonstrated that the TNF-α expression level in the annulus fibrosus is negatively related to VAS [34], suggesting that TNF-mediated inflammatory response is not parallel to the level of pain. Therefore, TNF-α inhibitors administered for anti-inflammation might not be sufficient to simultaneously control both leg pain and lower back pain. (3) It is difficult to relieve the pain of patients who had a long disease course. Animal experiments have revealed that treatment immediately after the nerve root injury could be effective, while drug administration 10 days after the injury is often ineffective because of the occurrence of neuropathic pain [43]. Therefore, in the trials enrolled in the present study, the pain of patients with a disease course longer than 10 days was difficult to reduce. (4) Pain is not a decisive factor for the selection of discectomy. In clinical practice, the selection of discectomy depends mainly on the functional status, particularly the degree of disability. Some scholars have stated that the disability level is unrelated to the acute or chronic pain [18]. Parameters such as the Oswestry Disability Index and the VAS are based on pain and thus cannot be used to assess the overall functional status of patients. In addition, Korhonen *et al.* [27] stated that there is no direct correlation between the selection of discectomy and the Oswestry Disability Index.

Interestingly, we found that TNF-α inhibitors could reduce the RR of discectomy or radicular block (combined endpoint) at medium-term follow-up, but not at short-term and long-term follow-up. Regarding this phenomenon, our explanations are as follows: (1) the neuroprotective role of TNF-α inhibitors cannot been fulfilled within a short period of time; (2) we conservatively speculated that TNF-α-related inflammation and neurotoxicity are not the major pathophysiological mechanisms of sciatica; thus, TNF-α inhibitors might not exhibit clinical value at long-term follow-up; (3) the bias of drug administration approaches might be involved in this result. Although the currently available evidence is not sufficient to determine the superiority of local injection and systematic drug administration, the efficacy of intravenous injection is dubious. The sensitivity analysis demonstrated that after the exclusion of three trials in which the drugs were administered through intravenous injection [11], [16], [27], TNF-α inhibitors significantly decreased the risk of discectomy or radicular block (combined endpoint; p = 0.049; Figure 6B). In addition, Ohtori *et al.* [12] stated their negative opinion regarding the use of intravenous injection. Moreover, the ineffectiveness of intravenously administered steroids was also indicated in other studies [44].

Regarding the finding that TNF-α inhibitors reduced the risk of discectomy or radicular block (combined endpoint) but did not increase the percentage of global perceived effect (satisfaction) or return to work (combined endpoint), we believe a possible explaination could be as following: the endpoint, global perceived effect (satisfaction) or return to work (combined endpoint), is related to the patients’ self-perceptions (such as pain), while discectomy or radicular block (combined endpoint) is related more closely to the patients’ functional status, particularly in the case of discectomy.

It is worth noting that in this study, we could not draw a definitive conclusion regarding the appropriate selection of the drug dose and the frequency of drug administration based on the current available evidence. Because the endpoint data of both the treatment and control groups changed proportionately (10 times), the differences in the VAS score ranges among the enrolled trials would not affect the quantitative analysis and result interpretation in the meta-analysis using SMD.

Compared with the study by Williams *et al.* [10], the present study has the following differences: (1) This study used SMD for the meta-analysis of endpoints that had a different score range, such as VAS-leg pain and VAS-lower back pain. (2) The meta-analysis of discectomy or radicular block (combined endpoint) was conducted using the follow-up data from different time points, i.e. short-term, medium-term and long-term, to evaluate the treatment outcomes more precisely. (3) This study discovered that TNF-α inhibitors cannot provide additional pain relief at all follow-up periods, but they can reduce the RR of discectomy or radicular block (combined endpoint) at the medium-term follow-up.

The limitations of this study are as follows: (1) the sample size was small, and the follow-up durations were inconsistent; (2) the data showed a skewed distribution, and data expressed with the mean value and without a standard deviation (e.g. the trial of Okoro *et al.* [22]) could not be included in the quantitative analysis of measurement data; (3) although the majority of the included trials were double-blinded or triple-blinded, most of the evaluating parameters adopted in these trials were subjective, and thus the outcomes of natural disease course or the medication treatment could not be distinguished; (4) most of the included trials only showed the results of the treatment analysis rather than the intention-to-treat (ITT) analysis.

**Conclusion:** According to the currently existing evidence, other than reducing the RR of discectomy of the radicular block (combined endpoint) at the medium-term follow-up, TNF-α inhibitors have limited clinical value in the treatment of sciatica caused by disc herniation and/or spinal stenosis.
